# Decreased lipid levels in adult with congenital heart disease: a systematic review and Meta-analysis

**DOI:** 10.1186/s12872-023-03455-w

**Published:** 2023-10-27

**Authors:** Fengdie Ma, Peiqiang Li, Shasha Zhang, Wenjing Shi, Jing Wang, Qinglong Ma, Meie Zhao, Ziyan Nie, Handan Xiao, Xinyi Chen, Xiaodong Xie

**Affiliations:** 1 Institute of Genetics, School of Basic Medical Sciences, Lanzhou University, Lanzhou, Gansu China; 2 The first people’s hospital of Lanzhou city, Lanzhou, Gansu China

**Keywords:** Adult congenital heart disease, Dyslipidemia, TC, HDL-C, LDL-C

## Abstract

**Background:**

Metabolic disorders were a health problem for many adults with congenital heart disease, however, the differences in metabolic syndrome-related metabolite levels in adults with congenital heart disease compared to the healthy population were unknown.

**Methods:**

We collected 18 studies reporting metabolic syndrome-associated metabolite levels in patients with congenital heart disease. Data from different studies were combined under a random-effects model using Cohen’s d values.

**Results:**

The results found that the levels of total cholesterol (Cohen’s d -0.68, 95% CI: -0.91 to -0.45), high-density lipoprotein cholesterol (Cohen’s d -0.63, 95% CI: -0.89 to -0.37), and low-density lipoprotein cholesterol (Cohen’s d -0.32, 95% CI: -0.54 to -0.10) were significantly lower in congenital heart disease patients compared with controls. Congenital heart disease patients also had a lower body mass index (Cohen’s d -0.27, 95% CI: -0.42 to -0.12) compared with controls. On the contrary, congenital heart disease patients had higher levels of hemoglobin A1c (Cohen’s d 0.93, 95% CI: 0.17 to 1.70) than controls. Meanwhile, there were no significant differences in triglyceride (Cohen’s d 0.07, 95% CI: -0.09 to 0.23), blood glucose (Cohen’s d -0.12, 95% CI: -0.94 to 0.70) levels, systolic (Cohen’s d 0.07, 95% CI: -0.30 to 0.45) and diastolic blood pressure (Cohen’s d -0.10, 95% CI: -0.39 to 0.19) between congenital heart disease patients and controls.

**Conclusions:**

The lipid levels in patients with congenital heart disease were significantly lower than those in the control group. These data will help in the health management of patients with congenital heart disease and guide clinicians.

**PROSPERO registration number:**

CRD42022228156.

**Supplementary Information:**

The online version contains supplementary material available at 10.1186/s12872-023-03455-w.

## Introduction

Congenital heart disease (CHD) is the most common cause of congenital abnormalities [1]. In recent years, significant advances in pediatric cardiology and cardiovascular surgery have improved the survival rates of CHD patients into adulthood and have greatly increased the number of adult patients with CHD [2]. Nevertheless, the heart malformation of most patients cannot be completely cured by surgery, and many patients continue to suffer from hemodynamic abnormalities [3], such as coronary artery replantation during transposition repair can lead to abnormal coronary blood flow reserve [4]. Adult CHD patients were also susceptible to cardiac-related complications, including heart failure, arrhythmia, endocarditis, cardiac conduit obstructions, thrombosis, aortic disease and pulmonary hypertension [5, 6]. As a result, the health condition of these patients needs to be monitored throughout their lives.

Metabolic syndrome (MS) usually includes a combination of any three of the following metabolic disorders: including obesity, dyslipidemia, hyperglycemia, and hypertension [7, 8]. Clinical evidence showed that the incidence of MS was higher in adults with CHD than in the general population, which means that adult CHD patients were at higher risk of metabolic disorders than the general population [9]. Autonomic dysfunction, which was more pronounced in the obese population, increases cardiovascular workload, hemodynamic stress, severe arrhythmias, and significant cardiac pathology, so obesity may complicate the management of patients with adult CHD who were already at risk for ventricular dysfunction, arrhythmias, and heart failure [10]. Hypertension may increase the risk of postoperative aortic dilatation in patients with CHD. Dellberg et al. reported that type 2 diabetes were more common in adults CHD population, and another study showed a higher prevalence of impaired fasting glucose in patients with CHD than in the general population [11]. Studies have indicated a higher prevalence of MS in patients with CHD. However, contrasting findings have been reported regarding lipid levels. Some studies have observed lower levels of total cholesterol (TC), high-density lipoprotein cholesterol (HDL-C), and low-density lipoprotein cholesterol (LDL-C) in acyanotic CHD (ACHD) patients compared to control groups [12–15]. On the other hand, cyanotic CHD (CCHD) patients did not have significantly lower levels of TC and LDL-C compared to controls, but they had significantly lower levels of HDL-C [16, 17]. Moreover, a separate study concluded that HDL-C levels in cyanotic CHD patients were significantly lower than in ACHD patients [12]. Dyslipidemia is a common manifestation of MS, and the identification and treatment of dyslipidemia are crucial for improving the overall health status in adults with CHD [12, 18, 19]. Abnormal metabolism was accompanied by an increased risk of cardiovascular disease (CVD), which was probably the leading cause of death in most adult CHD patients [20, 21]. Therefore, monitoring of lipids, BMI, glucose and blood pressure in CHD patients is beneficial for the management of health status in CHD patients. Studies have shown that patients with CHD are more likely to have obesity and hypertension compared to the general population [22, 23].

Despite this, there is still a lack of systematic review and meta-analysis of the differences in MS-related metabolite levels between adults with CHD and healthy controls. In this study, we systematically assessed the differences in blood lipid (TC, LDL-C, HDL-C, TG), HbA1c, glucose levels and blood pressure between adult CHD patients and the normal population through the meta-analysis. Our findings will help guide the future management of adult CHD and provide guidance for clinicians.

## Methods

### Search Strategy

We used PubMed [24] and Web of Science [25] to search the literature on metabolite levels of patients with CHD published before July 2022. Titles and abstracts were searched according to specific filters appropriate for the different databases. The main search terms used were [1] congenital heart disease, congenital heart defect, CHD, heart abnormality, heart malformation; and [2] adults; and [3] lipid, glucose, blood pressure and BMI. By screening the titles and abstracts (FD Ma and PQ Li), studies that meet the requirements were filtered out and evaluated by reading the full text. The guarantors of the review were XD Xie.

### Study selection

Studies on CHD published in English were included in the title and abstract screening stage. The full texts of all relevant literature were further evaluated. If the study was [1] English literature, [2] adult congenital heart disease, [3] case-control study or cohort study, [4] human study, and [5] reports data on metabolism-related indicators, including TC, HDL-C, LDL-C, triglycerides (TG), hemoglobin A1c (HbA1c), and glucose, as well as blood pressure data were included. Conversely, we excluded the following studies [1] Syndromic congenital heart disease (i.e. syndromic disorders associated with the development of congenital heart disease, such as Down syndrome, Marfan syndrome), [2] Ischemic heart disease and Coronary heart disease, [3] Maternal and child congenital heart disease, [4] reviews, conference papers, case reports, [5] published data were incomplete, [6] repeated publications.

### Data extraction

Evaluation and extraction of the data contained in the literature were performed independently by 2 authors and in consultation with the third author in case of disagreement. The following data were mainly extracted: authors, year of study publication, geographic area of the research, type of study design, the period for collecting cases, sample size, age, gender and body mass index (BMI) of the participant, whether the case group was operated or not, metabolite level data and classification of congenital heart disease.

### Study Quality assessments

The quality of studies included was guaranteed by using the Newcastle-Ottawa Scale (NOS), which is a tool recommended by the Cochrane Collaboration for observational studies to assess the risk of bias [26]. The star system with a range from zero to 9 stars was used to assess the quality of study [27]. The criteria included 8 items with a maximum of 9 stars. We included studies with a quality level above 6 stars for meta-analysis [28].

### Statistical analyses

In this meta-analysis, the method of standardized mean difference (i.e., Cohen’s d value) was used as the effect size to compare the differences in TC, HDL-C, LDL-C, TG, HbA1c, blood glucose levels, blood pressure and BMI between CHD patients and healthy controls under a random-effect model or a fixed-effect model. The units of metabolite levels reported in all studies were normalized to mg/dl before analysis. The normal control ranges for human lipid levels are: Total cholesterol, ideal value: <200 mg/dl; critical value: 200-239 mg/dl; excessive value: >240 mg/dl. Triglycerides, ideal value: <150 mg/dl; critical value: 150-199 mg/dl; excessive value: >200 mg/dl. HDL cholesterol, ideal value: >50 mg/dl; critical value: 35-50 mg/dl; risk value: <35 mg/dl; LDL cholesterol, ideal value: <130 mg/dl; critical value: 130-159 mg/dl; excess value: >160 mg/dl. Cohen’s d is a parameter used to compare the magnitude of the mean difference effect, which can indicate the magnitude of the difference between the overall means under different treatments and can be compared across studies. Cohen’s d values were considered a small effect size at 0.2, a moderate effect size at 0.5, and a large effect size at 0.8 [29]. The effect size and the corresponding 95% confidence interval (CI) were calculated by the online tool [30]-a practical meta-analysis effect size calculator used the mean ± standard deviation (SD) of related metabolite indicators reported in the literature. To determine which model to use, the heterogeneity between studies was assessed by the *P*-value and *I*^*2*^ statistics corresponding to the Cochran Q test. The *I*^*2*^ statistic was used to assess the percentage of total variation across studies that is due to heterogeneity rather than chance (*I*^*2*^ > 75% indicates high heterogeneity, 51–75% indicates substantial heterogeneity, 26-50% indicates moderate heterogeneity, and ≤ 25% indicates low heterogeneity). If *P*-value < 0.05 and *I*^*2*^ > 50%, the analysis was performed using a random-effects model, and if *P*-value > 0.05 and *I*^*2*^ < 50%, the analysis was performed using a fixed-effects model. To explore the source of heterogeneity, subgroup analysis was carried out by dividing the different geographical areas where the study was located. The symmetrical distribution of the funnel chart and the Egger’s test assessed publication bias (*P* < 0.05 indicates that the results were significant and there was publication bias). All statistical analyses were conducted in R software, version 4.0.4.

## Results

### Identification of studies

**Fig. 1:**
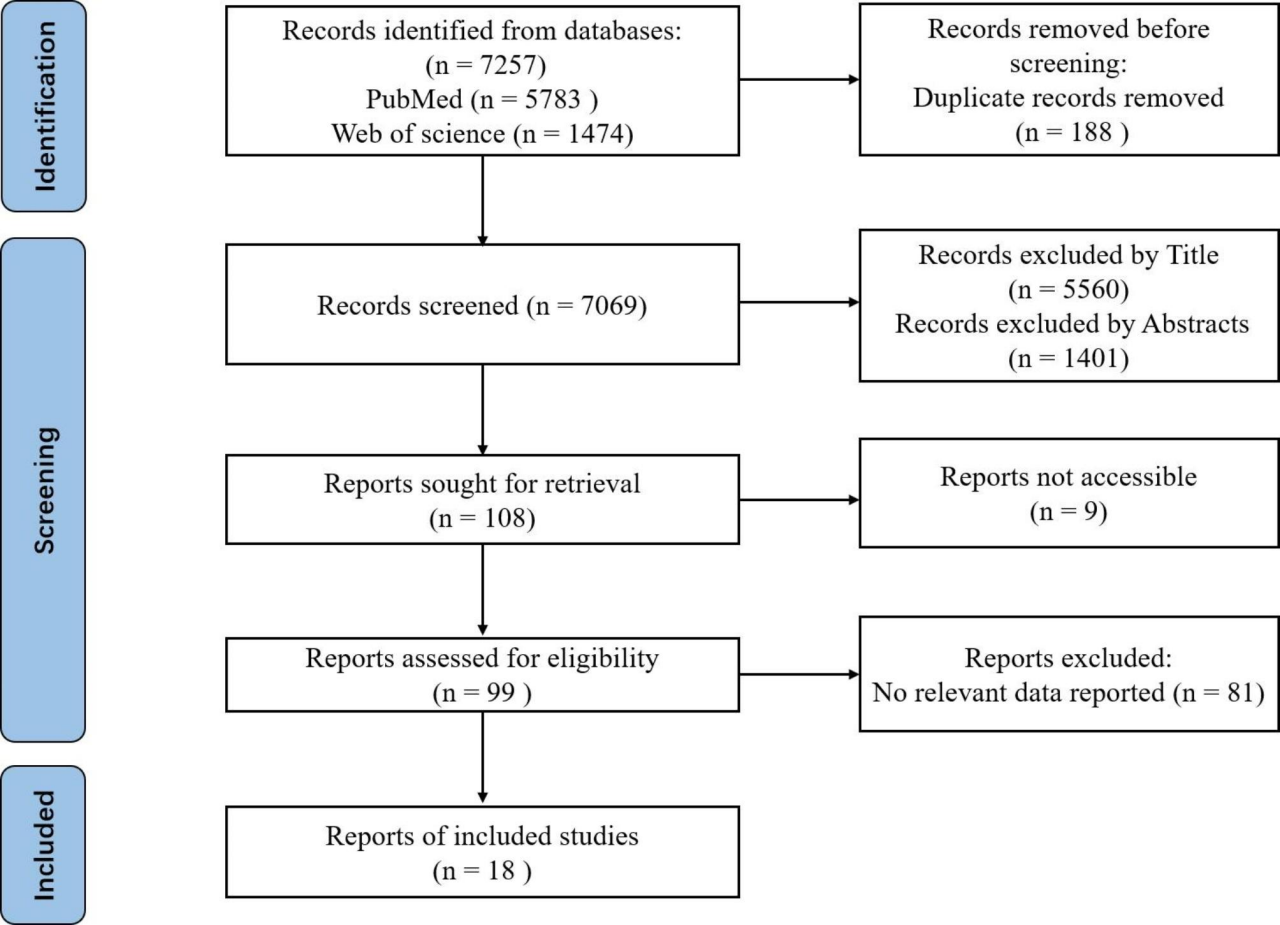
Flow chart of the literature search

At first, a total of 7257 articles meeting the requirements were collected with 5783 of them from PubMed and 1474 of them from the Web of Science. Among them, 188 duplicate studies were excluded, and then 6961 studies including reviews, conference papers, case reports, or studies that did not meet our inclusion criteria were excluded by reading the titles and abstracts. After reading and evaluating the full texts of the remaining 108 studies, 18 of them met the inclusion criteria and were included for meta-analysis (Fig. 1).

### Characteristics of studies

Eventually, the 18 studies, which involved 3613 CHD patients and 5664 healthy controls, met the inclusion and exclusion criteria and were included in this meta-analysis (Table 1). All literatures included were published between 2001 and 2021. There were 1993 male CHD patients and 1620 female CHD patients matched 2782 male controls and 2882 female controls. Almost all studies were based on adult CHD patients except for 5 studies including Santos 2001 (age 27.0 ± 12.0), Katayama 2006 (age 15.3 ± 11.9), Martínez 2010 and 2014 (selection criteria were 14 years and older), Jan 2015 (age 27.3 ± 12.1). A total of 13 studies reported the BMI of subjects, 10 studies were matched for BMI between cases and controls. Among the CHD patients, 637 were without surgery, 760 were treated with surgery, and 2216 were unclear whether they were treated with surgery or not. The candidate studies were 3 retrospective case-control studies, 3 prospective case-control studies, 1 observational case-control study, 1 Cross-sectional cohort study and 1 randomized case-control study. Besides these, 9 studies were conducted in Europe (Netherlands, Italy, Spain, Belgium, Sweden, Poland, Germany), 4 studies were conducted in Asia (Japan), 3 studies in the Americas (Brazil, United States), and 2 studies in Europe combined with Oceania (Denmark, Sweden, Norway, Australia).

**Fig. 2:**
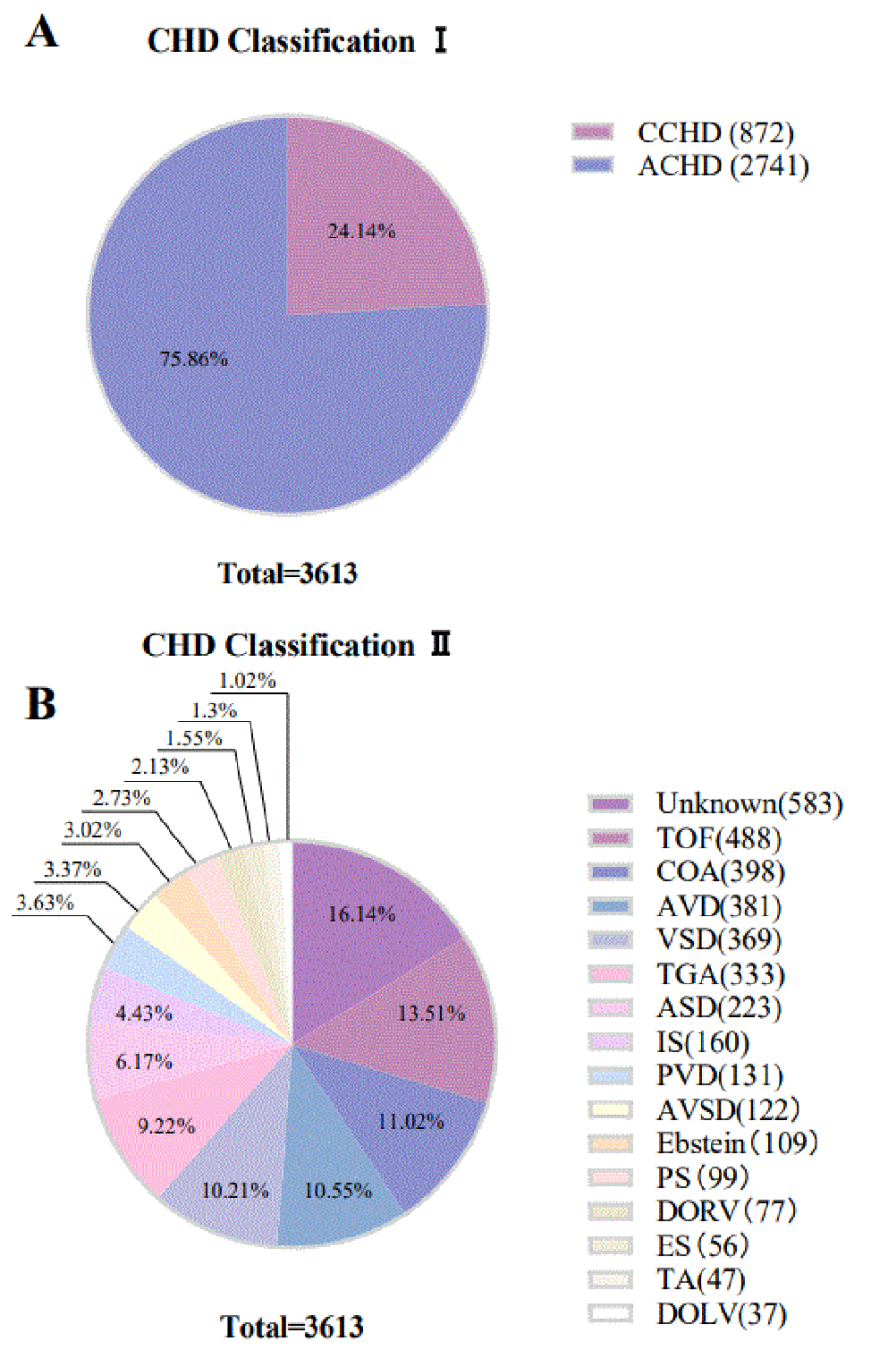
Classification of congenital heart disease. **(A)** CHD Classification I CCHD, cyanotic congenital heart disease; ACHD, acyanotic congenital heart disease. **(B)** CHD Classification II detailed types of CHD. TOF, Tetralogy of Fallot; COA, Coarctation of the aorta; AVD, Aortic valve disease; VSD, Ventricular septal defect; TGA, Transposition of the great arteries; ASD, Atrial septal defect; IS, Isolated shunts; PVD, Pulmonary valve disease; AVSD, Atrioventricular septal defect; PS, Pulmonary stenosis; DORV, Double outlet right ventricle; ES, Eisenmenger syndrome; TA, Tricuspid atresia; DOLV, Double outlet left ventricle

In terms of all CHD patients, there were 872 patients with CCHD, accounting for 24.14%, and 2741 patients with ACHD, accounting for 75.86% (Fig. 2A). For the detailed phenotypes, tetralogy of Fallot (TOF), coarctation of the aorta (COA), aortic valve disease (AVD), and ventricular septal defect (VSD) were the 4 main phenotypes, accounted for 16.14%, 13.51%, 11.02%, and 10.55% respectively. Other phenotypes include transposition of the great arteries (TGA), atrial septal defect (ASD), pulmonary valve disease (PVD), atrioventricular septal defect (AVSD), double outlet of the right ventricle (DORV), Eisenmenger syndrome (ES), etc. (Fig. 2B).

**Table 1:** Selected characteristics of 18 studies included in the meta-analysis

First author	Year	Geographic region	Type of study design	Study period	Sample size (case/control)	Age (case/control)	Gender (case/control)	Surgery	Analytical indicators	CHD classification
Santos	2001	Brazil	Case-control study	Not reported	41/48	27.0 ± 12.0 / 28.0 ± 11.0	F27; M14 /F32; M16	No surgery	TC; TG	Classification I CCHD,41. Classification II VSD,13; ASD,7; DORV,6; AVSD,4; TGA,1; Others,10.
Engvall	2001	Sweden	Case-control study	Not reported	18/36	36.0 ± 11.0 / 36.0 ± 10.0	F9; M9 /F18; M18	Surgery:14 No surgery:4	Systolic BP; Diastolic BP	Classification I ACHD,18. Classification II CoA, 18.
Katayama	2006	Japan	Case-control study	Not reported	18/27	15.3 ± 11.9 /15.3 ± 9.0	F5; M13 /F15; M12	Unknown	TC; HDL-C; LDL-C; TG; HbA1c	Classification I CCHD,18.
Andrzej	2007	Poland	Case-control study	Not reported	14/13	32.0 ± 3.0 /32.0 ± 3.0	F8; M6 /F9; M4	Unknown	BP systolic; BP diastolic; BMI	Classification I CCHD, 14. Classification II VSD 3, ES 9, TOF, 2.
Ohuchi	2009	Japan	Prospective case-control study	2005.12-2008.10	16/27	30 ± 10 /27 ± 5	F10; M6 /F15; M12	No surgery	TC; LDL-C; HDL-C; TG; Glucose; HbA1c; BMI	Classification I CCHD,16.
Duffels	2010	Dutch; Italian	Observation case-control study	2007.3-2008.5	54/54	38(19–60) /37(18–60)	F24; M30 /F25; M29	No surgery	Glucose; TC; LDL-C; HDL-C; TG; BMI; BP systolic; BP diastolic	Classification I CCHD,54.
Martínez	2010	Spain	Randomized case-control study	Not reported	158/152	28.3(16.4–51.6) /33 (30–35)	F64; M94 /F103; M49	Surgery:82 No surgery:76	TC; LDL-C; HDL-C; TG; Glucose; BMI	Classification I CCHD,51; ACHD,107. Classification IIASD,11; VSD,25; TOF,17; CoA,16; AVSD,12; PS,12; TGA,14; AVD,6; PVD,4; Ebstein,4; DORV,4; Others,33.
Martínez	2014	Spain	Case-control study	Not reported	117/152	27.2 ± 10.8 /32.7 ± 1.8	F52; M65 /F103; M49	Unknown	TC; LDL-C; HDL-C; TG; BMI	Classification I CCHD,23; ACHD,94. Classification IIVSD,25; ASD,12; CoA,10; TGA,9; TOF ,8; PS,6; AVSD,7; AVD,4; Others,36.
Ohuchi	2014	Japan	Prospective case-control study	Not reported	38/27	32.0 ± 10.0 /27.0 ± 5.0	F19; M19 /F15; M12	No surgery:38	Glucose; HbA1c; BMI	Classification I CCHD, 38. Classification II TOF,10; VSD,4; DORV, 4; TGA,3; Others, 17.
Ju Ryoung	2015	Korea	Case-control study	2010.10-2011.4	90/135	48.4 ± 10.9/ 47.1 ± 10.3	F57; M33 /F76; M59	Surgery:90	TC; TG; HDL-C; LDL-C; BP systolic; BP diastolic	Classification I ACHD,90. classification II VSD,11; ASD,30; AVSD,5; TOF,22; DORV,4; Ebstein,6; PVD,3; TGA,3; TA,3; Other, 3.
Jan	2015	Germany	Cross-sectional cohort study	2011.6-2013.8	1125/322	27.3 ± 12.1/ 29.4 ± 18.4	F464; M661 /F165; M157	Unknown	Systolic BP; Diastolic BP; BMI	Classification I CCHD,54; ACHD, 1071. Classification II TGA, 213; TOF, 217; Ebstein, 66; PS, 51; COA, 127; AS, 189; IS, 121; others, 141.
First author	Year	Geographic region	Type of study design	Study period	Sample size (case/control)	Age (case/control)	Gender (case/control)	Surgery	Analytical indicators	CHD classification
Olga	2016	Poland	Case-control study	2014.6-2015.6	36/35	42.33 ± 16.3/ 39.6 ± 10.4	F19; M17 /F19; M16	Unknown	Systolic BP; Diastolic BP; TC; LDL-C; HDL-C; Glucose; BMI	Classification I CCHD,36. Classification IIVSD, 7; ASD, 2; PVD, 4; TOF, 5; Ebstein, 4; others, 14.
Flannery	2017	United States	Retrospective case-control study	Not reported	248/744	50.6 ± 9.2 /51 ± 9.1	F120; M128 /F360; M384	Unknown	TC; HDL-C; LDL-C; BMI; Systolic BP, Diastolic BP	Classification I CCHD,79; ACHD,169. Classification II CoA,91; TOF,57; IS,39; Ebstein,20; TGA,15; ES,7; Others,19.
Tarp	2018	Denmark; Sweden; Norway; Australia	Case-control study	2014.8-2018.2	74/74	49.5(23–78) /50(24–78)	F42; M32 /F42; M32	Unknown	TC; LDL-C; HDL-C; TG; Hb1Ac; BMI; Systolic BP, Diastolic BP	Classification I CCHD,74. Classification IIVSD,40; ASD,11; TOF,7; DORV,3; AVSD,3; Others,10.
Martínez	2019	Spain	Retrospective case-control study	2008.1-2018.9	818/1955	33(25–41) /30(22–42)	F358; M460 /F903; M1052	Surgery:410 No surgery:408	TC; LDL-C; HDL-C; TG; Glucose	Classification I CCHD,192; ACHD, 626. Classification II AVD,96; PVD,102; ASD,90; VSD,139; CoA,63; PS,12; TOF,66; Ebstein,8; AVSD,48; TGA,45; DORV,14; ES,40; Others, 107.
Mahmoud	2019	Belgium	Retrospective case-control study	2013.6-2015.5	539/1737	32.0 ± 9.3/ 38.7 ± 10.7	F249; M290 /F897; M840	Unknown	BMI	Classification I CCHD, 155; ACHD, 384. Classification IIVSD, 78; ASD, 54; PS, 30; PVD, 14; AVD, 86; COA, 73; TOF, 74; AVSD, 34; TGA, 30; DORV, 21; Others, 45.
Tarp	2020	Denmark; Sweden; Norway; Australia	Case-control study	Not reported	45/45	50(47–55) /52(44–57)	F24; M21 /F24; M21	Unknown	TC; LDL-C; HDL-C; TG; Hb1AC; BMI; Systolic BP, Diastolic BP	Classification I CCHD,5; ACHD,40. Classification IIVSD,24; ASD, 6; AVSD,3; TOF,3; DORV,2; Ebstein,1; PVD,1; Others,5.
Lubert	2021	United States	Prospective case-control study	2012.3-2019.5	164/81	30.3(22.8–34.4) /34.8(23.9–44.4)	F69; M95 /F61; M20	Surgery:164	TC; LDL-C; HDL-C; BMI	classification I CCHD,22; ACHD,142. classification II DOLV, 37; DORV, 19; PVD,3; AVSD,6; TA, 43; Other, 56.

Gender: F, female; M, male

TC, Total cholesterol; HDL-C, High-density lipoprotein cholesterol; LDL-C, Low-density lipoprotein cholesterol; TG, Triglycerides; HbA1c, hemoglobin A1c; BP, blood pressure

CCHD, Cyanotic congenital heart disease; ACHD, Acyanotic congenital heart disease

VSD, Ventricular septal defect; ASD, Atrial septal defect; DORV, Double outlet right ventricle; DOLV, Double outlet left ventricle; AVSD, Atrioventricular septal defect; TGA, Transposition of the great arteries; TOF, Tetralogy of Fallot; CoA, Coarctation of the aorta; PS, Pulmonary stenosis; AVD, Aortic valve disease; PVD, Pulmonary valve disease; TA, Tricuspid atresia; IS, Isolated shunts; ES, Eisenmenger syndrome

### Assessment of Study Quality

The quality of all studies was evaluated by using the Newcastle-Ottawa scale (Table 2). All literature included ranks above 6 stars, indicating the high quality of the studies and meeting the requirements of meta-analysis. 88.9% of studies (16/18) provided a clear case definition and underwent independent validation of the case in the original studies. Subjects included were diagnosed at least 2 times or based on at least 2 diagnostic methods, including echocardiography and/or cardiac magnetic resonance and/or cardiac catheterization; or by looking up original records such as hospital medical records. The cases collected in all studies were representative, eligible CHD patients were collected within a specified time, or all cases came from a specific hospital. Controls in 66.7% of studies (12/18) were selected from normal communities, and another 33.3% of studies also clearly defined selected controls. In 15 studies, the case and control groups were matched for sex and/or age. A total of 13 studies reported data on lipid levels. For the measurement of blood lipid levels, 6 studies did not report detailed methods, 3 studies used spectrophotometry, 2 studies used enzymatic colorimetry, and the other 2 studies used laboratory or hospital standard methods.

**Table 2:** Overview of research quality assessment

Study	Is the case definition adequate?	Representativeness of the cases	Selection of Controls	Definition of Controls	Comparability	Ascertainment of exposure	Same method of ascertainment for cases and controls	Non-Response Rate	Total
Santos 2001	*	*		*	**	*	*		7
Engvall 2001	*	*	*	*	*	*	*		7
Katayama 2006		*		*	**	*	*		6
Andrzej 2007	*	*		*	*	*	*		6
Ohuchi 2009	*	*	*	*		*	*		6
Duffels 2010	*	*	*	*	**	*	*		8
Martínez 2010		*	*		**	*	*	*	7
Martínez 2014	*	*	*		**	*	*	*	8
Ohuchi 2014	*	*	*	*		*	*		6
Ju Ryoung 2015	*	*		*	**	*	*		7
Jan 2015	*	*	*	*		*	*		6
Olga 2016	*	*		*	*	*	*		6
Flannery 2017	*	*		*	**	*	*		7
Tarp 2018	*	*	*	*	**	*	*		8
Martínez 2019	*	*	*		**	*	*		7
Mahmoud 2019	*	*	*	*	*	*	*		7
Tarp 2020	*	*	*		**	*	*		7
Lubert 2021	*	*	*	*	*	*	*		7

Note: For items 1–4 (selection) and items 6–8 (exposure), the research can earn up to 1 star (*). Item 5 (comparability) can provide up to 2 stars (**)

### The levels of TC, HDL-C, LDL-C were significantly lower in CHD patients

**Fig. 3:**
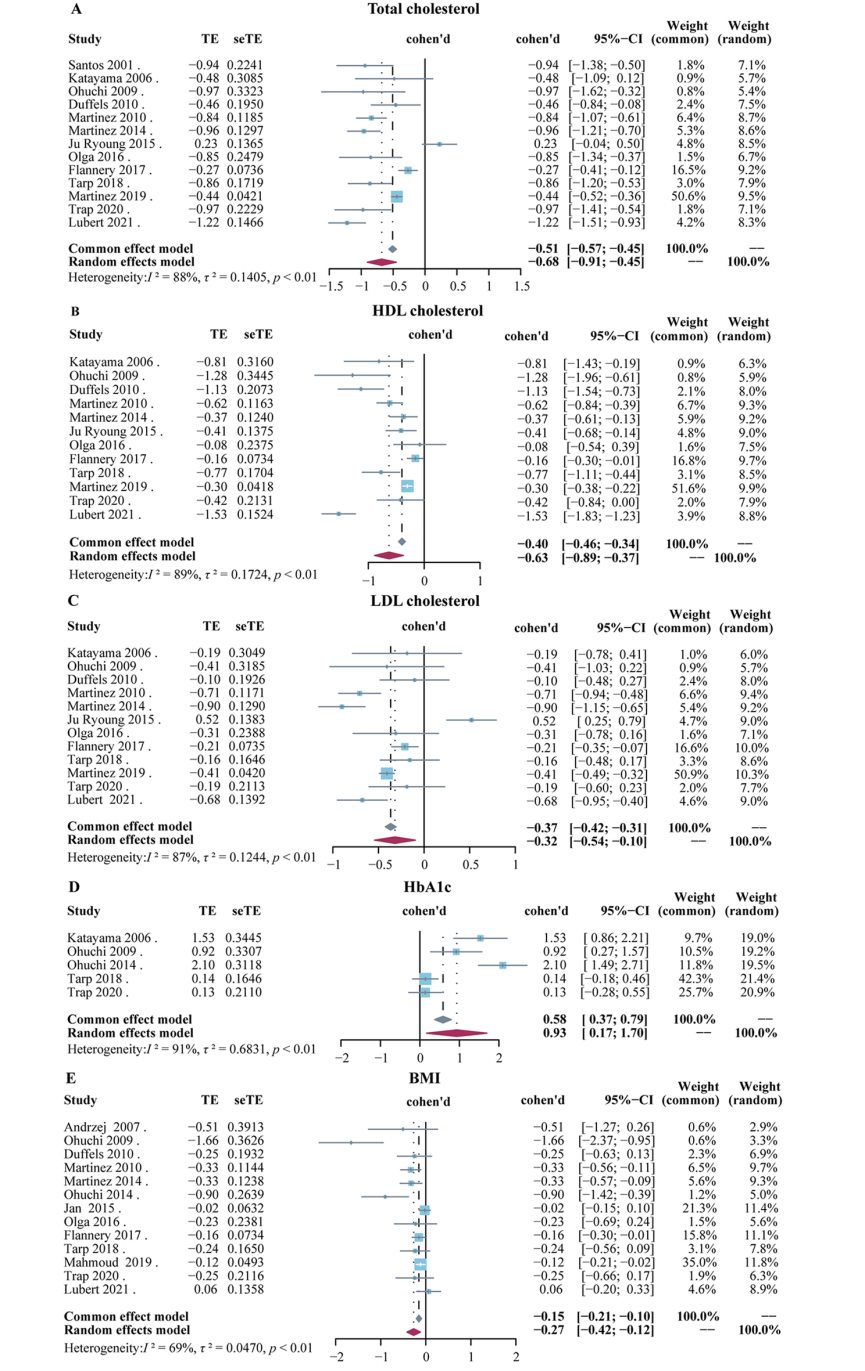
Forest plot of blood lipid levels between CHD patients and healthy controls. **(A)** Total cholesterol; **(B)** High-density lipoprotein cholesterol; **(C)** Low-density lipoprotein cholesterol; **(D)** HbA1c; **(E)** BMI. These studies were listed by year of publication. The data was expressed as a Cohen’s d value. The blue square represents the Cohen’s d value of a single study, the gray diamond represents the Cohen’s d value from the fixed-effects model meta-analysis, and the red diamond represents the Cohen’s d value from the random-effects model meta-analysis. The horizontal line represents 95% CI. Abbreviation: CI, confidence interval

The meta-analysis demonstrated that the levels of TC, HDL-C, and LDL-C in CHD patients were significantly lower than controls. The Cohen’s d value for TC calculated by meta-analysis was − 0.68 with a range of -1.22 to 0.23 (95% CI: -0.91 to -0.45), *I*^*2*^ = 88%, *P* < 0.01 (Fig. 3A). The Cohen’s d range of HDL-C was − 1.53 to -0.08, and the combined Cohen’s d value was − 0.63 (95% CI: -0.89 to -0.37), *I*^*2*^ = 89%, *P* < 0.01(Fig. 3B). The results showed a significant medium effect size on decreased the levels of TC and HDL-C, compared with the control groups. Similar results were obtained for LDL-C levels, which were also reduced in CHD patients [combined Cohen’s d value: -0.32(95% CI: -0.54 to -0.10), *I*^*2*^ = 87%, *P* < 0.01] (Fig. 3C). As far as HbA1c was concerned, there were only 5 studies. Compared with controls, the levels of HbA1c were higher in CHD patients. The combined Cohen’s d was 0.93 (95% CI: 0.17 to 1.70), *I*^*2*^ = 91%, *P* < 0.01 (Fig. 3D). We also found that CHD patients had significantly lower BMI than controls, although the combined Cohen’s d value had only a small effect size [combined Cohen’s d value: -0.27 (95% CI: -0.42 to -0.12]), *I*^*2*^ = 69%, *P* < 0.01] (Fig. 3E).

**Fig. 4:**
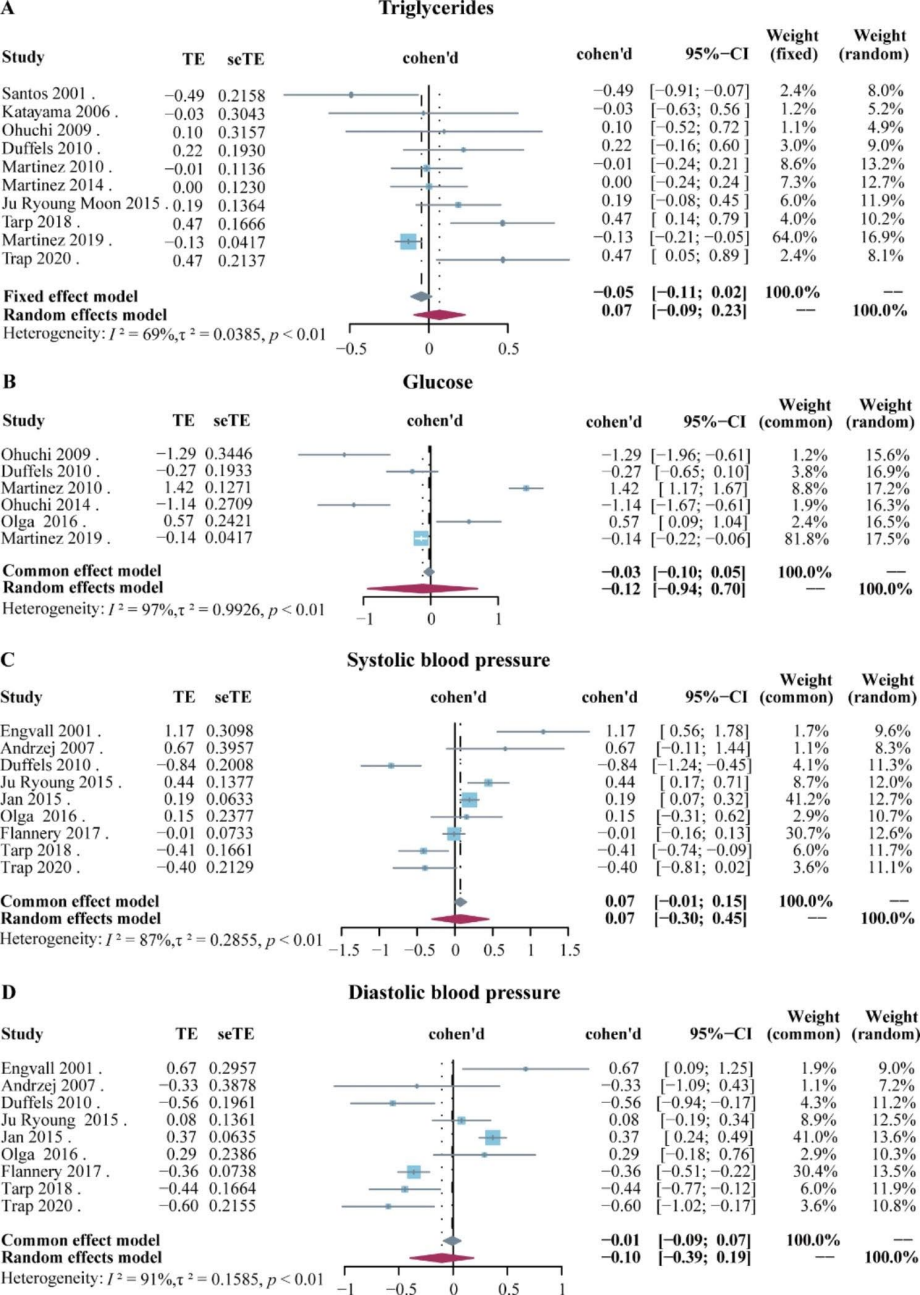
Forest plot of metabolites levels and blood pressure between CHD patients and healthy controls. **(A)**Triglycerides; **(B)** Glucose; **(C)** Systolic blood pressure; **(D)** Diastolic blood pressure. These studies were listed by year of publication. The data was expressed as a Cohen’s d value. The blue square represents the Cohen’s d value of a single study, the gray diamond represents the Cohen’s d value from the fixed-effects model meta-analysis, and the red diamond represents the Cohen’s d value from the random-effects model meta-analysis. The horizontal line represents 95% CI. Abbreviation: CI, confidence interval

Differently, there were no significant differences in TG, blood glucose levels, diastolic and systolic blood pressure between CHD patients and controls. Cohen’s d values for TG levels combined were 0.07 (95% CI: -0.09 to 0.23), *I*^*2*^ = 69%, *P* < 0.01 (Fig. 4A). The combined Cohen’s d values for blood glucose levels reported in the 6 articles were − 0.12 (95% CI: -0.94 to 0.70), *I*^*2*^ = 97%, *P* < 0.01 (Fig. 4B). The combined Cohen’s d value for systolic blood pressure was 0.07 (95% CI: -0.30 to 0.45), *I*^*2*^ = 87%, *P* < 0.01 (Fig. 4C). Cohen’s d values for diastolic blood pressure combined were − 0.10 (95% CI: -0.39 to 0.19), *I*^*2*^ = 91%, *P* < 0.01 (Fig. 4D).

### Sensitivity analysis and publication Bias

To explore whether the heterogeneity between different studies was attributable to the inclusion of extreme research results, we conducted a sensitivity analysis. The results were presented in Supplementary Fig. 1. Sensitivity analyses were performed separately for the different biomarkers, and each biomarker was analyzed by excluding one study in turn and using the remaining studies, the overall results did not change significantly, indicating that there were no extreme phenomena in the studies included. In the analysis of publication bias, Egger’s test showed no evidence of publication bias for TC (*P* = 0.1308) (Supplementary Fig. 2A), LDL-C (*P* = 0.6761) (Supplementary Fig. 2C), TG (*P* = 0.095) (Supplementary Fig. 2E), HbA1c (*P* = 0.0633) (Supplementary Fig. 2G), Glucose (*P* = 0.8673) (Supplementary Fig. 2I) levels, and diastolic (*P* = 0.5120) (Supplementary Fig. 2K) and systolic blood pressure (*P* = 0.8641) (Supplementary Fig. 2M). Also, no publication bias was observed by visual inspection of funnel plots (Supplementary Fig. 2B, 2D,2 F, 2 H, 2 J, 2 L and 2 N). But for HDL-C levels (*P* = 0.0453) (Supplementary Fig. 3A and 3B) and BMI (*P* = 0.0118) (Supplementary Fig. 3D and 3E), both Egger’s test and funnel chart showed that there may be publication bias (*P* < 0.05). Nonetheless, further analysis using the trim and fill method showed that the comprehensive effect size of HDL-C (Cohen’s d = -0.3201) and BMI (Cohen’s d = -0.1115) were consistent with the results before the analysis (Supplementary Fig. 3C and 3 F), which could be explained to some extent, the comprehensive effect size was not be affected by publication bias in HDL-C levels and BMI.

### Subgroup Analysis by Geographic Region

We found varying degrees of heterogeneity among the studies, so further subgroup analyses were performed. Subgroup analyses were performed according to different geographical regions, including the Americas, Asia, Europe, and Europe combined with Oceania. This is because there are differences in genetic factors and dietary habits in different geographic regions, both of which can have an impact on lipid levels. In terms of TC levels, the CHD patients from the America (Cohen’s d, -0.79; 95% CI: -1.37 to -0.21; *I*^*2*^ = 95%), Europe (Cohen’s d, -0.70; 95% CI: -0.93 to -0.46; *I*^*2*^ = 83%), and Europe combined with Oceania (Cohen’s d, -0.90; 95% CI: -1.17 to -0.64; *I*^*2*^ = 0%) were significantly lower than controls, but not in Asians (Cohen’s d, -0.36; 95% CI: -1.06 to 0.35; *I*^*2*^ = 85%) (Supplementary Fig. 4A). The HDL-C levels of CHD patients from Asia (Cohen’s d, -0.76; 95% CI: -1.27 to -0.25; *I*^*2*^ = 67%), Europe (Cohen’s d, -0.49; 95% CI: -0.80 to -0.18; *I*^*2*^ = 82%), and Europe combined with Oceania (Cohen’s d, -0.62; 95% CI: -0.96 to -0.28; *I*^*2*^ = 40%) were significantly lower than control groups, while the HDL-C levels of the Americans (Cohen’s d, -0.84; 95% CI: -2.18 to 0.51; *I*^*2*^ = 98%) was not significantly different between CHD patients and the control groups (Supplementary Fig. 4B). LDL-C levels of CHD patients was significantly lower than control groups only in Europe (Cohen’s d, -0.51; 95% CI: -0.78 to -0.24; *I*^*2*^ = 81%). There were no significant differences in LDL-C levels between CHD patients from Asia (Cohen’s d, 0.03; 95% CI: -0.56 to 0.62; *I*^*2*^ = 80%), America (Cohen’s d, -0.43; 95% CI: -0.89 to 0.03; *I*^*2*^ = 89%), and Europe combined with Oceania (Cohen’s d, -0.17; 95% CI: -0.42 to 0.09; *I*^*2*^ = 0%) and the controls (Supplementary Fig. 5A). The relationship between TG levels and CHD was complicated in different populations. TG levels were decreased in American (Cohen’s d, -0.49; 95% CI: -0.91 to -0.07) patients and increased in Europe combined with Oceania (Cohen’s d, 0.47; 95% CI: 0.21 to 0.73; *I*^*2*^ = 0%) patients. There were no significant differences in TG levels between CHD patients and controls in Asian (Cohen’s d, 0.14; 95% CI: -0.08 to 0.37; *I*^*2*^ = 0%) and European (Cohen’s d, -0.05; 95% CI: -0.17 to 0.07; *I*^*2*^ = 33%) (Supplementary Fig. 5B).

Additionally, the CHD patients in Asia had higher HbA1c levels and lower glucose levels compared with the control groups (Cohen’s d, 1.52; 95% CI: 0.85 to 2.20; *I*^*2*^ = 70% and Cohen’s d, -1.20; 95% CI: -1.61 to -0.78; *I*^*2*^ = 0%). The HbA1c levels in the Europe combined with Oceania (Cohen’s d, 0.14; 95% CI: -0.12 to 0.39; *I*^*2*^ = 0%) population and the glucose levels in the European populations (Cohen’s d, 0.39; 95% CI: -0.38to 1.16; *I*^*2*^ = 98%) were not significantly different between CHD patients and the control groups (Supplementary Fig. 6A and 6B). In terms of BMI, patients with CHD in Europe (Cohen’s d, -0.18; 95% CI: -0.31 to -0.06; *I*^*2*^ = 41%) and Asia (Cohen’s d, -1.24; 95% CI: -1.97 to -0.50; *I*^*2*^ = 65%) had a lower BMI, while in the Americas (Cohen’s d, -0.08; 95% CI: -0.28 to 0.13; *I*^*2*^ = 49%) and Europe combined with Oceania (Cohen’s d, -0.24; 95% CI: -0.50 to 0.01; *I*^*2*^ = 0%) had a BMI that did not differ from the control groups (Supplementary Fig. 6C). In European CHD patients, blood pressure was not significantly different from healthy controls (Systolic blood pressure: Cohen’s d, 0.23; 95% CI: -0.42 to 0.89; *I*^*2*^ = 89%; Diastolic blood pressure: Cohen’s d, 0.10; 95% CI: -0.34 to 0.55; *I*^*2*^ = 83%), but in Europe combined with Oceania populations CHD patients had lower blood pressure (systolic blood pressure: Cohen’s d, -0.41; 95% CI: -0.66 to -0.15; *I*^*2*^ = 0%; Diastolic blood pressure: Cohen’s d, -0.50; 95% CI: -0.76 to -0.24; *I*^*2*^ = 0%)( Supplementary Fig. 7A and 7B).

## Discussion

We investigated MS-related metabolite levels in 3613 CHD patients and 5664 controls in 18 studies. Although studies suggest that MS is more common in patients with CHD, our results contradict these findings. The results of this meta-analysis indicated that among CHD patients, lipid levels including TC, HDL-C, and LDL-C levels, and BMI were significantly lower than healthy controls. HbA1clevels were elevated in CHD patients, while triglyceride, glucose levels and blood pressure were not significantly different from healthy controls. To the best of our knowledge, this study is the first meta-analysis to evaluate the difference in MS-related metabolite levels between CHD patients and healthy controls, which can provide advantageous information for clinicians and CHD survivors, as well as guide the clinical treatment of CHD patients. Our results showed that CHD patients have lower lipid levels than controls. Meanwhile, other studies have shown an association between CHD and cardiovascular disease risk in later life. CHD patients are found to have a higher risk of developing cardiovascular disease, including stroke, heart failure, and coronary artery heart disease and more [28]. It has been demonstrated that mature cardiomyocytes take fatty acids as the primary substrates to generate ATP [31]. Thus, the reduced lipid levels might have a negative effect on energy metabolism of cardiomyocyte and potentially contribute to cardiac dysfunction, which might serve as one of the risk factors for increased cardiovascular risk in adult CHD. In the future, sophisticated basic and clinical studies will help to uncover how hypolipidemia affects the cardiomyocyte energy production and its association with cardiovascular risk.

The underlying causes of dyslipidemia in CHD patients were multifaceted. Surgical intervention was the primary consideration. There was evidence that CHD patients undergoing Fontan surgery would develop a series of liver abnormalities over time, including coagulopathy, cholestasis, liver fibrosis, cirrhosis, and hepatocellular carcinoma [32–34]. Thus, liver dysfunction and resulting dyslipidemia may lead to hypolipidemia in CHD patients [35]. Nevertheless, we also found that some patients in our studies without undergoing Fontan surgery still had a low level of blood lipid, suggesting that other factors also played a role in the abnormal lipid metabolism of CHD patients. It is noted that endocrine diseases caused by metabolic abnormalities often appear in adult CHD patients. For instance, the prevalence of subclinical hypothyroidism (SCH) in adult CHD patients (9.6%) is higher than in people without known thyroid diseases (4.6%) [36, 37]. For patients with CCHD, proteinuria is the result of long-term cyanosis [38]. Previous studies had shown that inflammation, proteinuria, and autoimmune diseases contribute to SCH, and that SCH was related to changes in serum cholesterol levels [39, 40]. Hence, the decline in metabolic capacity caused by SHC in CCHD patients may be one of the reasons for their abnormal blood lipid levels. Moreover, the low blood lipid levels of CHD patients may also be related to the decreasing iron storage levels [17, 41], because the low iron storage levels of CHD patients can reduce LDL-C [42]. Malnutrition is also a problem for patients with CHD, and different studies have reported inconsistent levels of malnutrition in patients with CHD. 85% of patients with CHD in the study by Tokel et al. were malnourished, which correlated with the patient’s household income and dietary intake. Results by Blasquez et al. showed that 15% of patients with CHD had moderate or severe malnutrition, with half of them exhibiting low caloric intake with little appropriate nutritional support [43–47]. Therefore, the undernutrition in CHD patients may also be one of the factors for their low levels of blood lipids. Our study also observed that the BMI of CHD patients was lower than healthy controls, which may be associated with developmental delay due to the lower weight gain during their childhood [48]. The lower BMI may partially explain the lower lipid levels of CHD patients, but it is unlikely that the direct determinant of lipid levels in CHD patients is body weight itself [12].

Genetic variants may also be associated with lipid levels. Apolipoprotein (APOB) and lipoprotein-related receptor protein 2 (LRP2) play roles in lipid metabolism as LDL apolipoprotein and transport of cholesterol, respectively [49, 50]. Rare mutations in *APOB* and *LRP2* inhibited the proliferation of cardiomyocytes and were associated with the occurrence of left heart hypoplasia syndrome (HLHS) [51]. These findings suggested that genetic variants in genes related to lipid metabolism may be responsible for affected lipid levels in CHD patients.

Our research did find differences in blood lipid levels of adult CHD between diverse populations. We considered that the living environment and diet were partially responsible for this difference. For example, adherence to the Mediterranean diet could reduce the prevalence of obesity (especially abdominal obesity) and MS [52, 53]. Studies have shown that energy expenditure levels were also associated with lipid levels in CHD patients, with more active young men showing lower TC and TG levels in comparison with their moderately active and sedentary peers [54]. Medication use may also affect blood lipid levels in CHD patients, such as beta-blockers lowering HDL-C levels [55]. Because of the lack of data on the correlation between living environment, dietary habits, energy expenditure levels and medication use with lipid levels in the included literature, we were unable to further analyze the relationship between the above factors and lipid levels in adult patients with CHD. To clarify whether the blood lipid levels of adult CHD patients were affected by these factors needs further study in the future.

Our results also showed that the HbA1c levels of CHD patients were significantly higher than healthy controls. A total of 5 studies reported HbA1c levels of 191 CHD patients, of which 175 were CCHD patients. Research had shown that long-term hypoxemia in CCHD patients could significantly increase the number of red blood cells, thereby prolonging the blood passage time and headed to a reduction in blood rheology [56]. Elevated levels of HbA1c may be related to the above results. In addition, it was reported that CCHD survivors have a significantly increased risk of type 2 diabetes (T2DM), which may also be related to their increased HbA1c levels [57]. Although studies have shown a higher prevalence of hypertension and diabetes in patients with CHD [11, 22], it is important to note that hyperglycemia can also lead to excessive production of superoxide beyond the mitochondrial electron transport chain via different molecular mechanisms. This, in turn, can result in vascular damage and the death of cardiomyocyte [58]. Therefore, the implementation of strict glycemic control through insulin therapy can be cardioprotective by enhancing glucose consumption as well as reducing both circulating levels and myocardial uptake of free fatty acids [59, 60]. However, no significant differences in blood glucose levels and blood pressure between patients with CHD and the general population were found in our study, which may be due to the limited data we collected and inconsistent measurement criteria for relevant biomarkers in the original studies, and more clinical data are needed in the future to elucidate the differences in blood glucose levels and blood pressure between patients with CHD and the general population.

There were some limitations in our study. First, for most results, there was a varying degree of heterogeneity between studies. This is because CHD comprises a spectrum of very different anatomical, physiological and clinical conditions. We hypothesize that the source of heterogeneity may be the specific classifications of CHD and whether surgical interventions were performed in CHD patients, which needs to be determined by more detailed clinical studies in the future. More importantly, the subgroup analysis revealed variations in the levels of relevant biomarkers across different populations. For example, Americans exhibited lower TG levels, although only one study was included. Conducting more clinical studies could help mitigate study bias and provide further insights into the variation in TG levels among CHD patients. Additionally, it can shed light on the impact of genetics and diet on their lipid levels in different regions.

Our study identified significantly lower lipid levels in patients with CHD than in control group. We hypothesize that low lipid levels pose a disadvantage in patients with CHD due to the adult heart’s heavy reliance on mitochondrial oxidative phosphorylation to produce ATP for energy [61], Cardiomyocytes, in particular, prefer lipids as their primary and more energy-efficient substrate, contributing to approximately 70% of total ATP production. This underscores the importance of plasma fatty acid uptake in maintaining cardiac viability [62]. These finding have implications for the health management of patients with CHD and can provide guidance for clinicians. However, further research is needed, both at the basic and clinical levels, are necessary to validate the impact of low-fat conditions on patients with CHD and explore the potential efficacy of nutritional interventions to mitigate this impact.

## Conclusions

The lipid levels in patients with congenital heart disease were significantly lower than those in the control group. These data will help in the health management of patients with congenital heart disease and guide clinicians.

### Electronic supplementary material

Below is the link to the electronic supplementary material.

Supplementary Material 1

Supplementary Material 2

## Data Availability

All data generated or analysed during this study are included in this published article [and its supplementary information files].

## Abbreviations

ACHD: acyanotic congenital heart disease
APOB: Apolipoprotein B
AVD: aortic valve disease
BMI: body mass index
CCHD: cyanotic CHD
CHD: congenital heart disease
CI: confidence interval
COA: coarctation of the aorta
CVD: cardiovascular disease
HbA1c: hemoglobin A1c
HDL-C: high-density lipoprotein cholesterol
HLHS: left heart hypoplasia syndrome
LDL-C: low-density lipoprotein cholesterol
LRP2: lipoprotein-related receptor protein 2
MS: metabolic syndrome
SCH: subclinical hypothyroidism
TC: total cholesterol
TG: triglyceride
TOF: tetralogy of Fallot
VSD: ventricular septal defect
