# Supplementary material for: Decreased lipid levels in adult with congenital heart disease: a systematic review and Meta-analysis
Source: BMC Cardiovasc Disord. 2023 Oct 27;23:523. doi: 10.1186/s12872-023-03455-w (PMC10612202; doi:10.1186/s12872-023-03455-w)
Supplement: Supplementary file 2 — Supplementary Material 2 [file 12872_2023_3455_MOESM2_ESM.docx]

**Supplementary Figure 1.** Sensitivity analysis results plot.


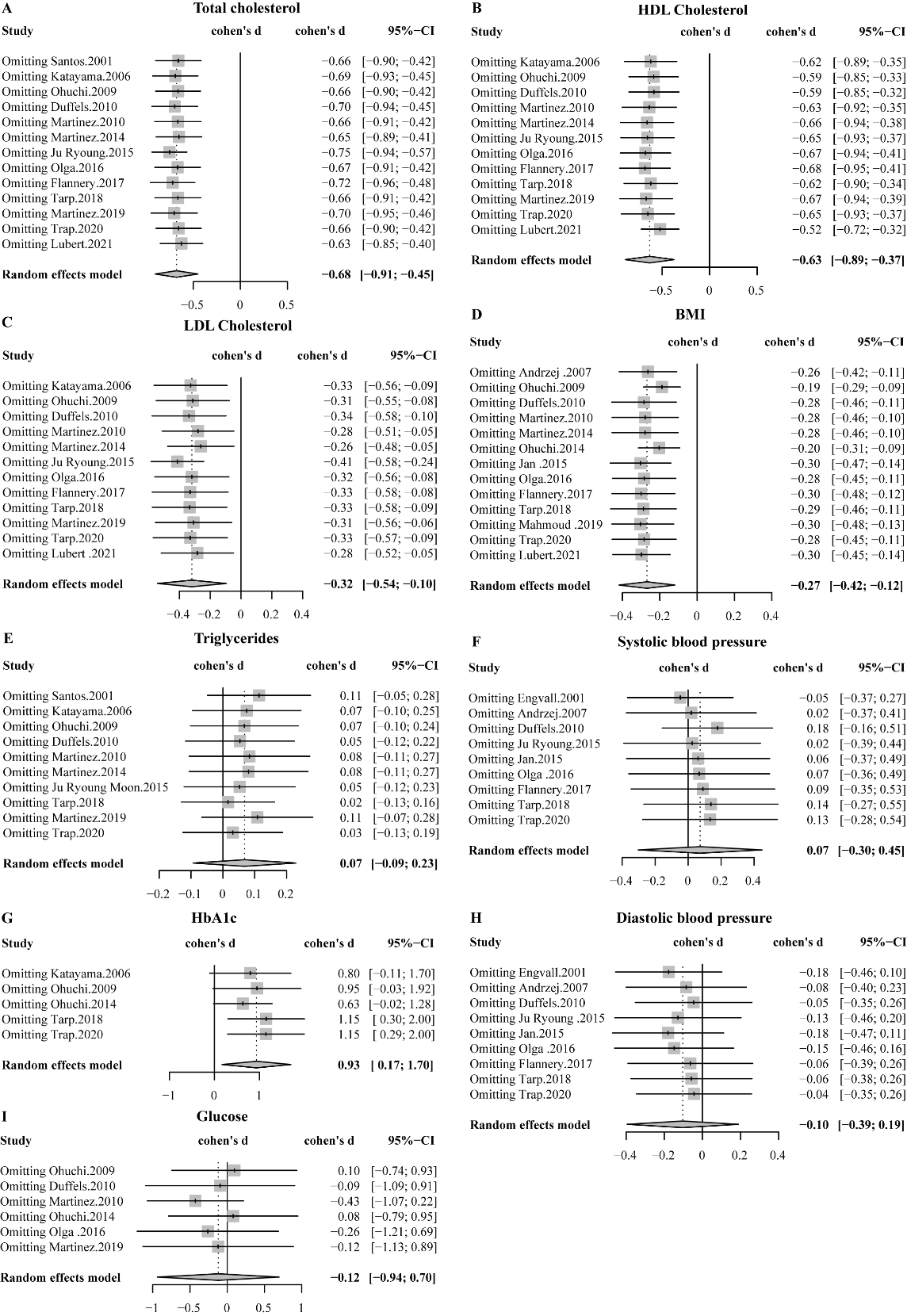


**Supplementary Figure 1.** Sensitivity analysis results plot. **(A)** Total cholesterol; **(B)** High-density lipoprotein cholesterol; **(C)** Low-density lipoprotein cholesterol; **(D)** BMI; **(E)** Triglycerides; **(F)** Systolic blood pressure; **(G)** HbA1c; **(H)** Diastolic blood pressure; **(I)** Glucose.

**Supplementary Figure 2.** Egger test plots for publication bias analysis of MS-related metabolite levels across studies.


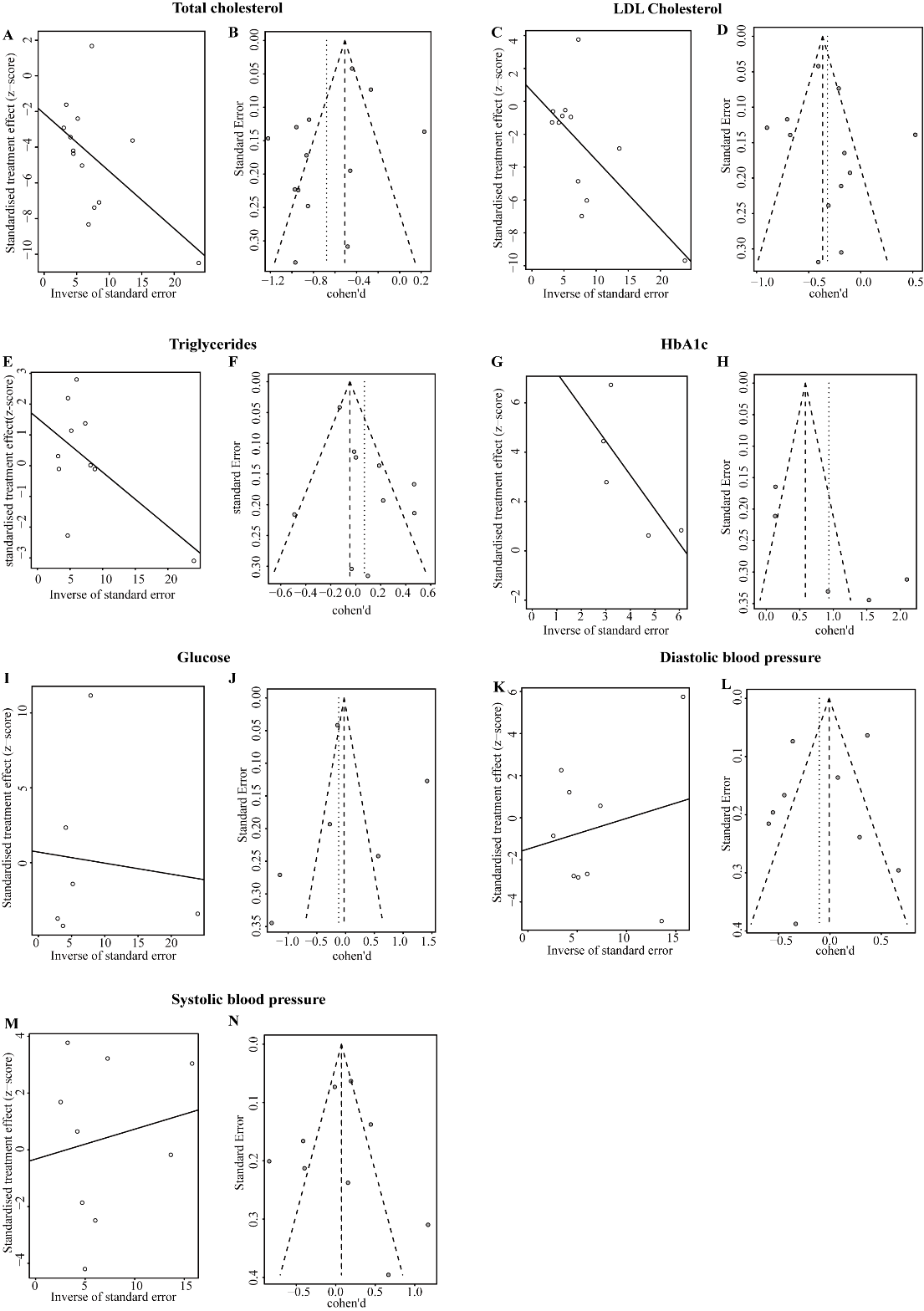


**Supplementary Figure 2.** Egger test plots for publication bias analysis of MS-related metabolite levels across studies. **(A)** Total cholesterol; **(C)** Low-density lipoprotein cholesterol; **(E)** Triglycerides; **(G)** HbA1c; **(I)** Glucose; **(K)** Diastolic blood pressure; **(M)** Systolic blood pressure. Funnel plots with 95% confidence limits for MS-related metabolite levels in patients with congenital heart disease. **(B)** Total cholesterol; **(D)** Low-density lipoprotein cholesterol; **(F)** Triglycerides; **(H)** HbA1c; **(J)** Glucose; **(L)** Diastolic blood pressure; **(N)** Systolic blood pressure.

**Supplementary Figure 3.** Egger test plots for publication bias analysis of MS-related metabolite levels across studies.


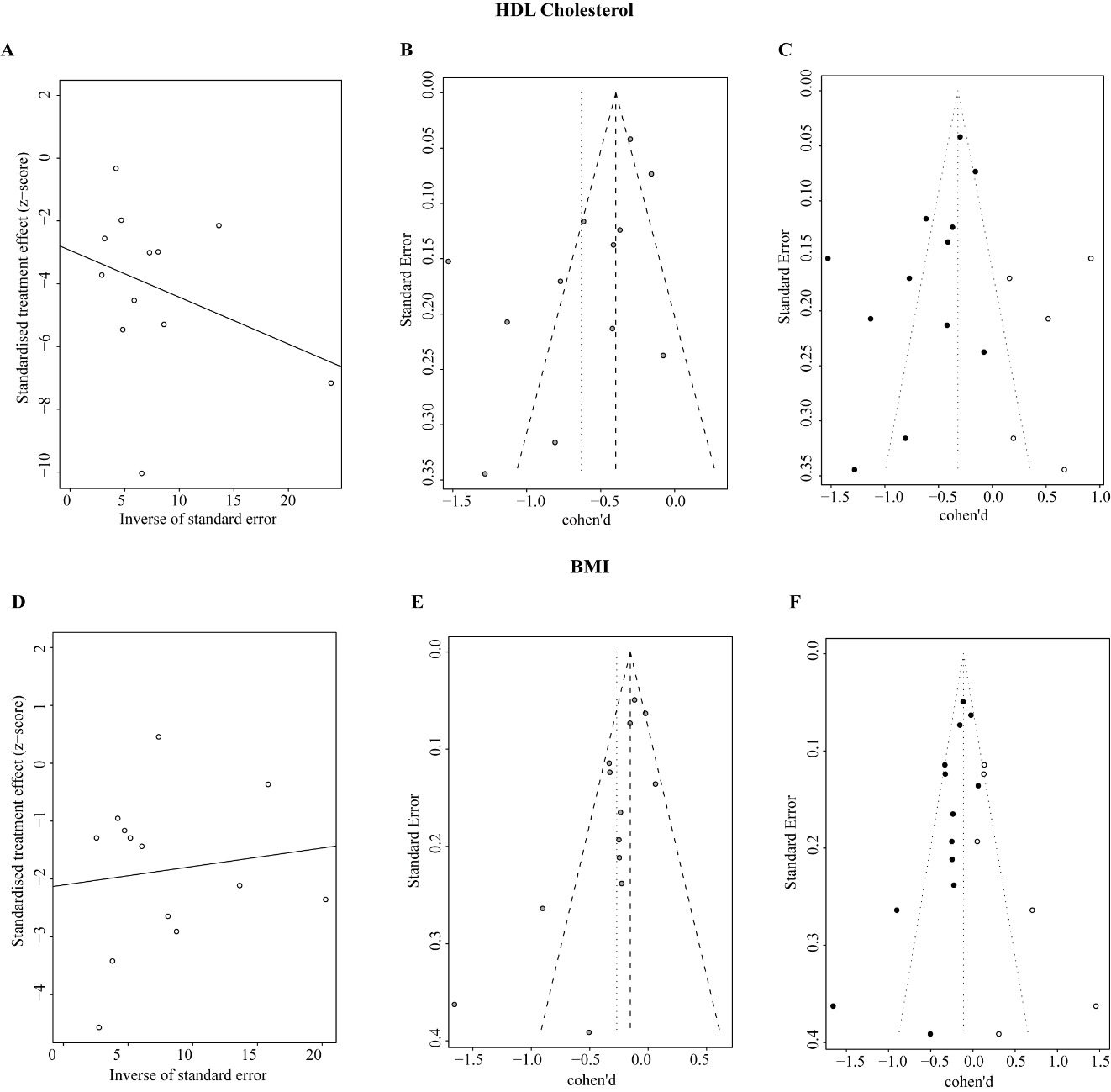


**Supplementary Figure 3.** Egger test plots for publication bias analysis of MS-related metabolite levels across studies. **(A)** High-density lipoprotein cholesterol; **(D)** BMI. Funnel plots with 95% confidence limits for MS-related metabolite levels in patients with congenital heart disease. **(B)** High-density lipoprotein cholesterol; **(E)** BMI. Funnel plot after trim-and-fill method analysis. Solid lines represent pooled effect estimates, and dashed lines represent pseudo-95% confidence limits. **(C)** High-density lipoprotein cholesterol; **(F)** BMI.

**Supplementary Figure 4.** Forest plot of subgroup analysis of blood lipid levels in CHD patients.


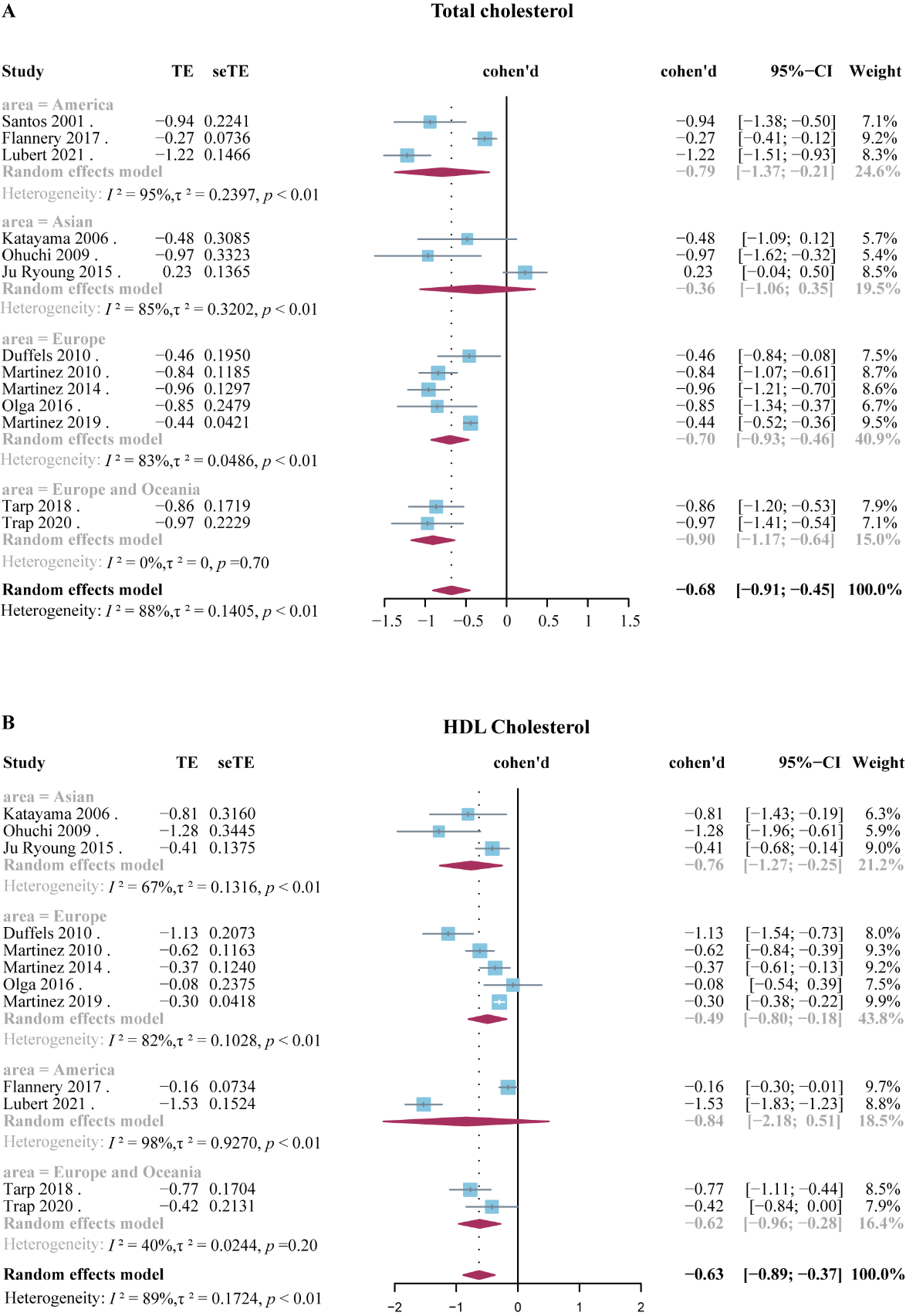


**Supplementary Figure 4.**  **(A)** Total cholesterol; **(B)** High-density lipoprotein cholesterol. These studies were listed by geographic area and year of publication. The data was expressed as a Cohen’s d value. The blue square represents the Cohen’s d value of a single study, the gray diamond represents the Cohen’s d value from the fixed-effects model meta-analysis, and the red diamond represents the Cohen’s d value from the random-effects model meta-analysis. The horizontal line represents 95% CI. Abbreviation: CI, confidence interval.

**Supplementary Figure 5.** Forest plot of subgroup analysis of blood lipid levels in CHD patients.


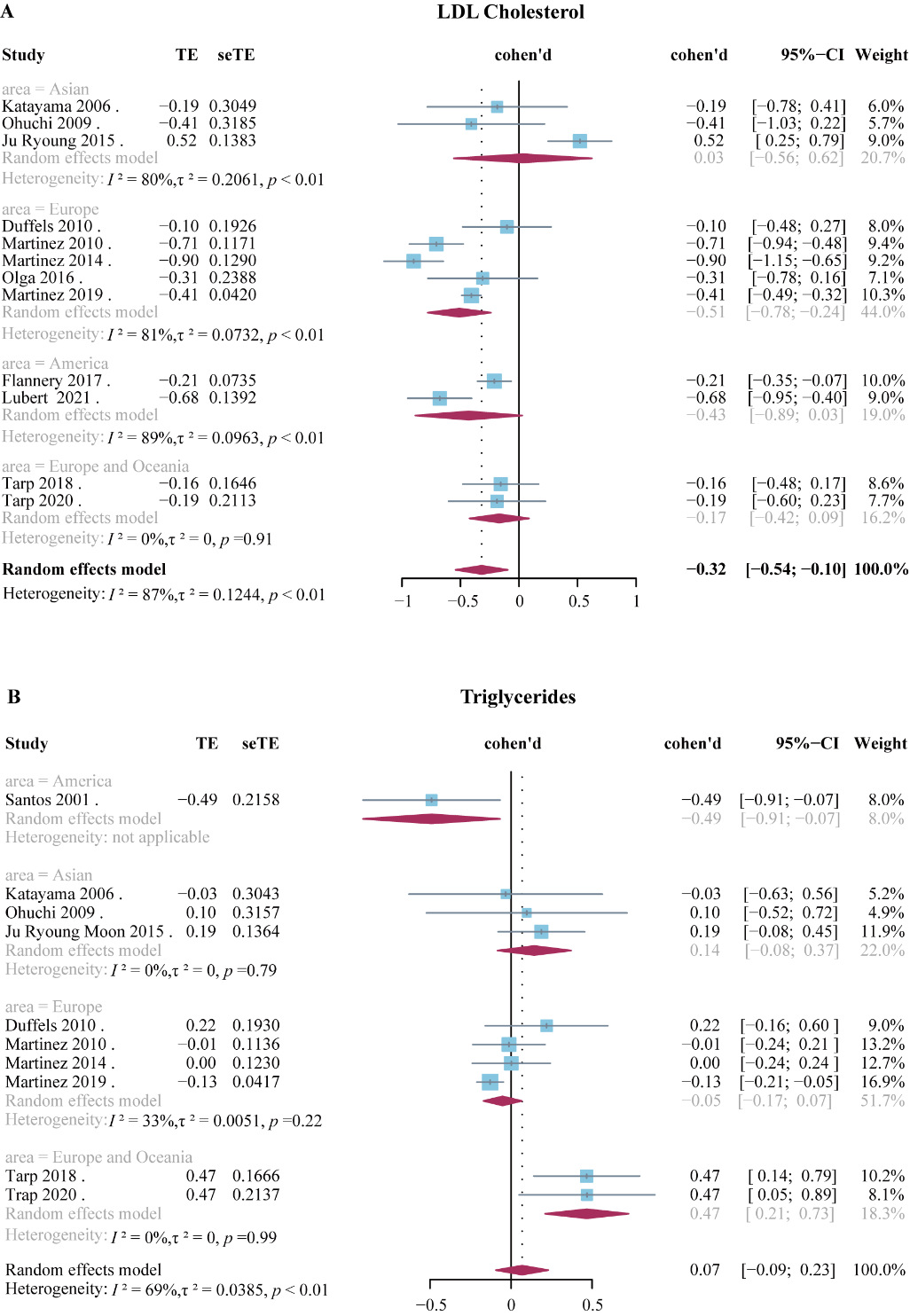


**Supplementary Figure 5. (A)** Low-density lipoprotein cholesterol; **(B)** Triglycerides. These studies were listed by geographic area and year of publication. The data was expressed as a Cohen’s d value. The blue square represents the Cohen’s d value of a single study, the gray diamond represents the Cohen’s d value from the fixed-effects model meta-analysis, and the red diamond represents the Cohen’s d value from the random-effects model meta-analysis. The horizontal line represents 95% CI. Abbreviation: CI, confidence interval.

**Supplementary Figure 6.** Forest plot of subgroup analysis of metabolites levels and BMI in CHD patients.


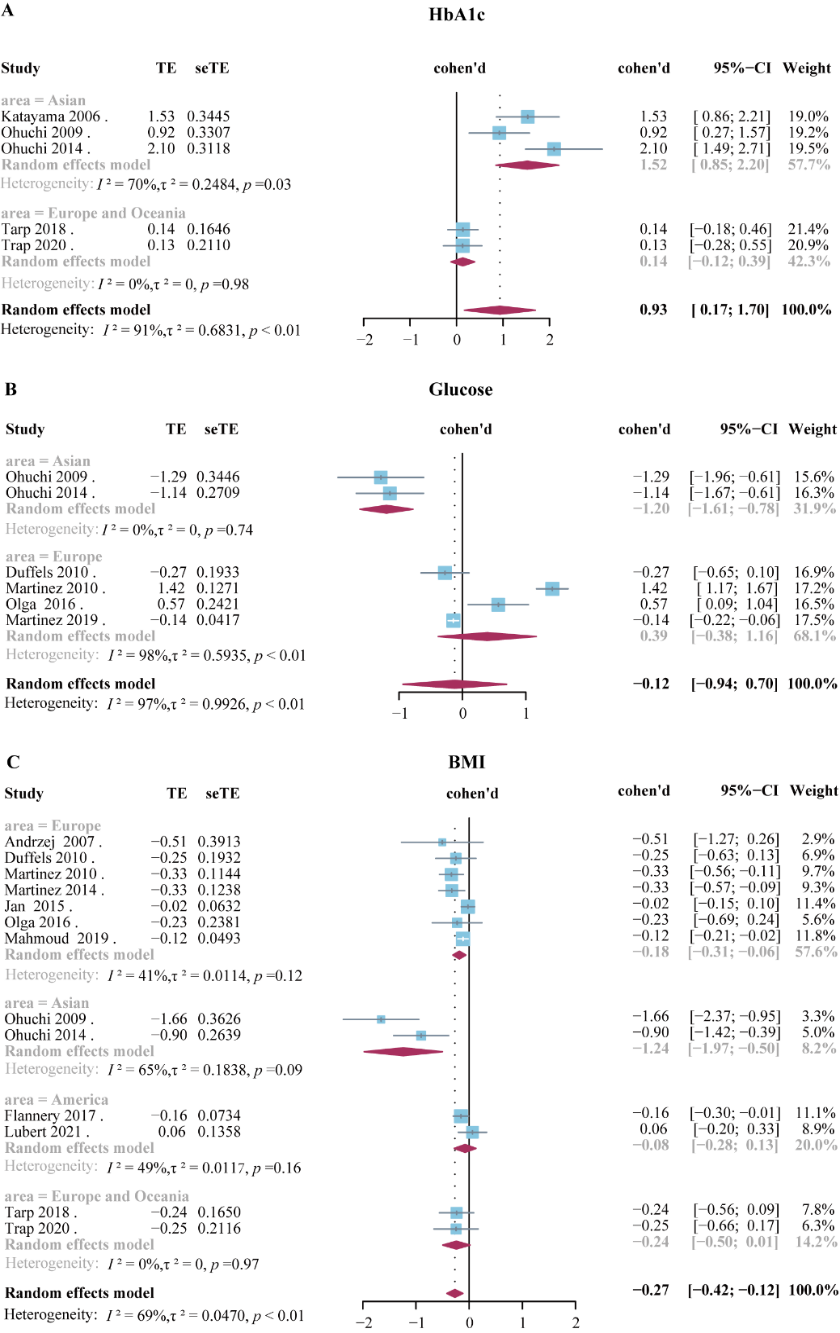


**Supplementary Figure 6.** **(A)**HbA1c; **(B)** Glucose; **(C)** BMI. These studies were listed by geographic area and year of publication. The data was expressed as a Cohen’s d value. The blue square represents the Cohen’s d value of a single study, the gray diamond represents the Cohen’s d value from the fixed-effects model meta-analysis, and the red diamond represents the Cohen’s d value from the random-effects model meta-analysis. The horizontal line represents 95% CI. Abbreviation: CI, confidence interval.

**Supplementary Figure 7.** Forest plot of subgroup analysis of blood pressure in CHD patients.


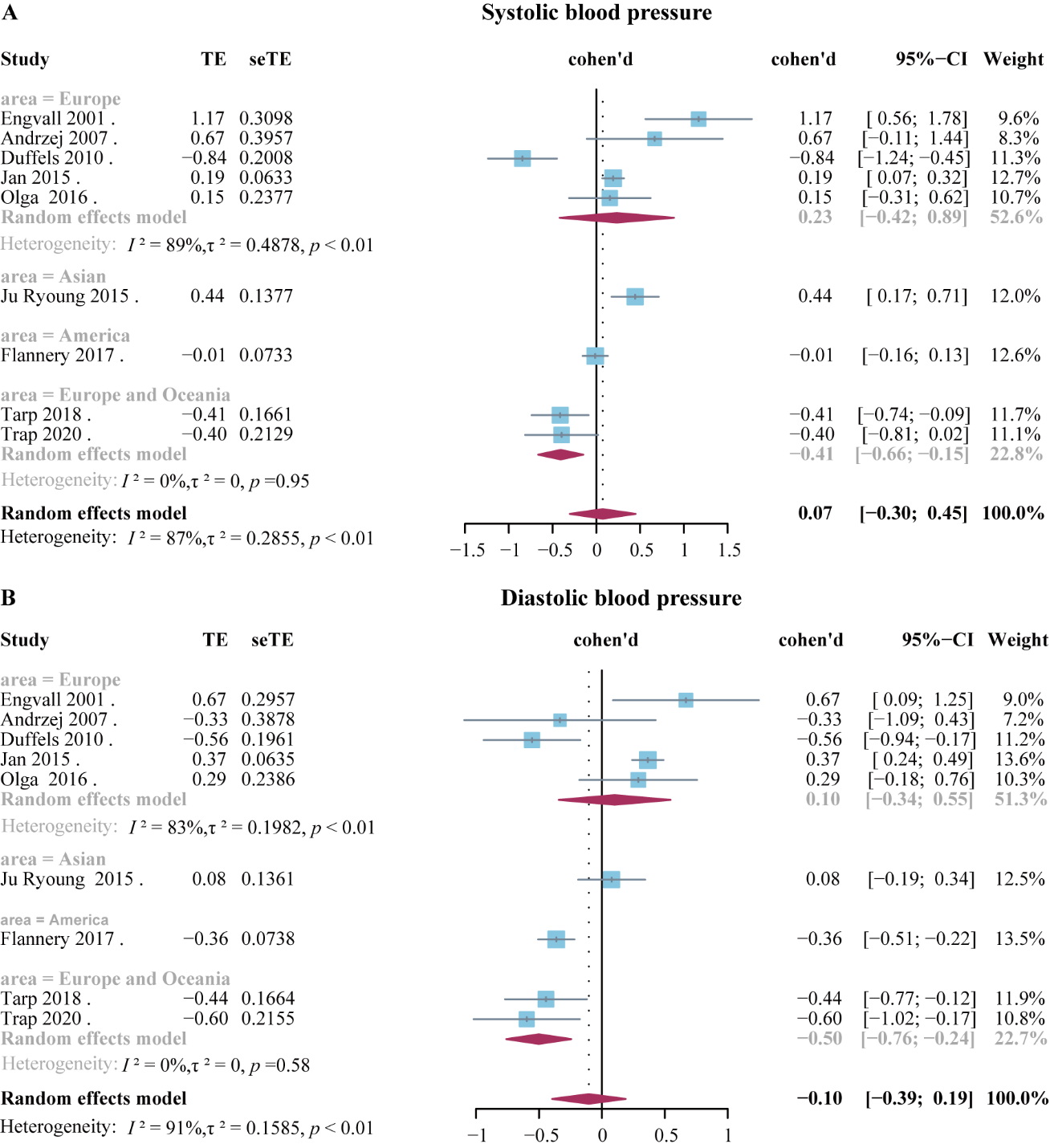


**Supplementary Figure 7. (A)** Systolic blood pressure; **(B)** Diastolic blood pressure. These studies were listed by geographic area and year of publication. The data was expressed as a Cohen’s d value. The blue square represents the Cohen’s d value of a single study, the gray diamond represents the Cohen’s d value from the fixed-effects model meta-analysis, and the red diamond represents the Cohen’s d value from the random-effects model meta-analysis. The horizontal line represents 95% CI. Abbreviation: CI, confidence interval.
